# Differentiation of Cotton Fabrics Using Stable Isotope Ratio Mass Spectrometry

**DOI:** 10.1021/jasms.6c00162

**Published:** 2026-08-31

**Authors:** Jason D. Eckmann, Douglas J. Beussman

**Affiliations:** St. Olaf College, Northfield, Minnesota 55057, United States

## Abstract

White cotton fibers are of special importance in forensics due to bleached cotton’s ubiquity in the clothing industry. While these fibers are often difficult to distinguish using conventional chemical techniques, stable isotope ratio mass spectrometry has proven a useful tool for their analysis. Our goal was to investigate the potential for applying this technique to the differentiation of t-shirts and other cotton products. Analysis was performed using an elemental analyzer, a high-temperature conversion/elemental analyzer, and a continuous flow isotope ratio mass spectrometer. Cotton samples of various manufacturing origins were analyzed to investigate the homogeneity and differentiability of the samples. Additionally, fibers of samples originating from the same source were studied. Differences between sewing thread and cloth in clothing samples were also analyzed. Differentiability was determined using ANOVA tests, and visualization was achieved using three-dimensional trivariate plot analysis. Each individual cotton sample was found to be largely homogeneous, but samples from different sources were distinguishable. Sewing thread and cloth fibers were also found to be differentiable. Based on this pilot study, stable isotope ratio mass spectrometry shows promise as a tool for the analysis of cotton fibers, especially when multiple isotopes are used to discriminate between samples.

## Graphical abstract

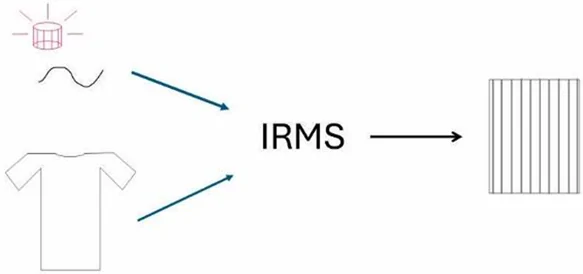

## 1 Introduction

White cotton fibers are of special importance to forensic analysis due to bleached cotton’s ubiquity in the clothing industry and potential use in criminal activity. Therefore, it is important to analyze these materials for any differentiating characteristics that could be used to connect a suspect with a fiber found at a crime scene. In the past, various methods have been used to identify and analyze cotton fibers. Infrared (IR) spectroscopy can discriminate cotton from other fabrics with similar appearances.^1,2^ Further IR and Raman spectroscopy analysis can differentiate fibers with different dyes and contaminants.^3^ However, these techniques were unable to distinguish between different white or bleached cotton fibers. Therefore, without the differentiating chemical and visible characteristics (such as color, texture, etc.) that allow for the differentiation of dyed cotton fibers,^4,5^ different methods need to be used to distinguish between white bleached cotton samples. Oxygen stable isotope analysis has been shown to have potential for the analysis of money.^6^ Research suggests that using stable isotope ratio mass spectrometry (IRMS) to analyze the ratios of stable isotopes in bleached cotton fibers, it is possible to distinguish between samples based on their geographic origins.^7^ Recently, sources of stable isotope fractionation in fibers including environmental conditions, biological source, and industrial processing, were reviewed.^8^ Stable isotope analysis has also shown promise for the ability to differentiate unprocessed cotton originating in different geographic locations.^9^

IRMS can differentiate between these cotton samples because the percent abundances of given elements (i.e., the ratios of heavy to light stable isotopes) differ under various conditions.^10^ Variations in stable isotope abundances of light elements are caused by processes that favor one isotope over another, a process called fractionation. Carbon isotope variations in plants, such as cotton, reflect the isotope composition of the reactant (CO_2_) in the ambient atmosphere around the plant, the diffusional isotope fractionation of CO_2_ into the plant, and a large biochemical/enzymatic isotope fractionation. The oxygen and hydrogen isotope composition of plant tissues typically have strong geographic correlations, reflecting largely geographic patterns of stable isotope ratios of source water to the plant (typically in the form of precipitation in which the isotope composition is affected by fractionation associated with progressive condensation of water from the atmosphere). The plant tissue stable isotope composition is further modified by processes such as soil water evaporation, transpiration and the biochemical processes associated with biosynthesis, all of which respond to local environmental conditions. Furthermore, stable isotope ratios can change with time, due to shifting water supplies and other environmental factors. Fractionation may also occur as a result of manufacturing and processing, leading to even more variation of stable isotopic signatures.^10,11^

Many other types of substances have also been shown to have differing ratios of stable isotopes based on geographic location, ranging from olive oil and wine to human hair and illicit drugs.^12–16^ More specifically, cotton has been shown to have differing stable isotope ratios based on growth stage and region of the plant, as described above.^17–19^ Processed cotton has also been analyzed and shown to have different stable isotopic signatures based on geographic origin.^7^

In this pilot study, we extended this research on processed cotton to investigate the stable isotope ratios of carbon, oxygen, and hydrogen in commercial items that had been previously worn or used such as cotton t-shirts, towels, and gauze. We analyzed eight cotton samples of various known and unknown origins. We also examined three undershirts of the same manufacturing origin, as well as the differences between cotton fiber and sewing/stitching thread from the same shirt to test for homogeneity of the shirts. With the small sample size of this study, the results should be viewed as a proof-of-concept of the analytical method.

## 2 Experimental Section

### 2.1 Materials

Three shirts were obtained for analysis. Shirt A, a men’s Dockers undershirt. Shirt B was a men’s Stafford undershirt. and Shirt C, a boy’s Hanes undershirt. Two cotton gauze pads, Gauze A and Gauze B, were obtained from Dukal Corp. and Dynarex Corp., respectively. Cloth A, a sample bolt of Bleached Mercerized Cotton Twill manufactured by TestFabrics, Inc., was also obtained for analysis. Two different towels of unknown origins were also used as further sources of manufactured cotton.

### 2.2 Preparation of Samples

Cotton samples were equilibrated under the same atmospheric conditions for 72 h to allow for equal deuterium exchange. Samples were then weighed into tin and silver capsules for ^13^C and ^2^H/^18^O analysis, respectively, and introduced into an autosampler, which was sealed and purged with helium to avoid atmospheric contamination. For ease of use, roughly 0.200 mg (approximately 1 mm × 1 mm) of each sample was cut and placed into each capsule. To demonstrate that smaller forensic samples can be used, samples of roughly 0.200 mg, 0.100 mg, 0.050 mg, and 0.020 mg were cut from the same sample and analyzed. No statistical differences in stable isotope ratio results were observed in the 0.200 to 0.050 mg samples, with some differences observed in the 0.020 mg samples. Thus, at least for our samples, the smallest sample that should be used was determined to be 0.050 mg (approximately 1 mm of a single thread).

The results of the trials are expressed as δ-values, which are numerical representations of the ratio of the heavier isotope to the lighter isotope of a given element in comparison with international stable isotope standards: VPDB (Vienna Pee Dee Belemnite) for ^13^C analysis and VSMOW (Vienna Standard Mean Ocean Water) for ^2^H and ^18^O analysis. These values are derived from eq 1, where *R*_sample_ is the ratio of heavier to lighter isotopes in the sample, and *R*_standard_ is the same measured ratio of the corresponding reference material.^10,20^

1 $$\delta =\frac{R_{\mathrm{sample}}}{R_{\mathrm{standard}}}-1$$

### 2.3 ^13^C Stable Isotope Analysis

Samples (∼0.200 mg) were weighed out and placed into tin capsules for analysis of their carbon stable isotope ratios. The samples were analyzed using a Costech ECS 4010 elemental analyzer (EA) in combination with a ThermoFisher Scientific Delta V Advantage isotope ratio mass spectrometer. The EA contained a combustion tube (1020 °C) that was filled with quartz wool, chromium oxide catalyst, and silvered cobaltous/cobaltic oxide, as well as a reduction tube (650 °C) filled with quartz wool and copper wire. To remove water from the postcombustion gases, a water trap filled with magnesium perchlorate was used. Two reference materials, sucrose (IAEA-CH-6, δ^13^C = −10.45 ± 0.03‰; NIST, Gaithersburg, MD) and asparagine (δ^13^C = −24.45 ± 0.16 ‰; in house standard), were used for two-point normalization of data within each sample sequence run.

### 2.4 ^2^H and ^18^O Stable Isotope Analysis

Samples (∼0.200 mg) were weighed into silver capsules for ^2^H and ^18^O analysis. A Thermo/Finnigan high temperature conversion elemental analyzer (TC/EA) coupled to a ThermoFisher Scientific Delta V Advantage isotope ratio mass spectrometer was used to analyze the samples. The ceramic reactor tube in the TC/EA was held at 1450 °C, and was packed with a graphite tube, glassy carbon tube with glassy carbon granulate, and silver and quartz wool. The temperature of the GC column was set to 90 °C with a He flow rate of 95 mL/min. Two keratin standards, kudu horn (KHS, δ^2^H = −35.3 ± 1.1 ‰, δ^18^O = 21.21 ± 0.17 ‰; USGS, Reston, VA) and caribou hoof (CBS, δ^2^H = −157.0 ± 0.9 ‰, δ^18^O = 2.39 ± 0.13 ‰; USGS, Reston, VA), as well as coumarin (δ^2^H = 82.3 ± 1.2 ‰; Indiana University, Bloomington, IN) were used for normalization of the ^2^H data within each sample sequence run, with benzoic acid 602 (BA602, δ^18^O = 71.28 ± 0.36 ‰; IAEA, Vienna, Austria) replacing coumarin for normalization of the ^18^O data. The kudu horn standards were used as check standards to verify instrument performance with the other standards used for two-point normalization. It should be noted that the presented δ^2^H values do not represent true nonexchangable hydrogen values and thus the reported values should not be compared to other results not part of this study. As all hydrogen measurements were collected under the same conditions and at the same time, a comparison of the results with the study presented here is still valid for determining differentiability between samples.

### 2.5 Experiments

#### 2.5.1 Eight Cotton Types

Eight different cotton samples were analyzed. Four fibers from each sample were taken from different regions of the source to check for homogeneity and to ensure the data was representative of the whole sample. A total of three batches were run over the course of 3 days, for a total of 12 data points from each cotton sample.

#### 2.5.2 T-Shirts of Identical Origins

Three shirts from the same batch and packaging were analyzed for homogeneity. Two different regions of each shirt were tested, with three fibers from each region being analyzed for a total of six samples per shirt.

#### 2.5.3 Sewing/Stitching Thread Versus Cloth

Three samples of sewing thread from three different regions of stitching (9 total), as well as three samples of cloth each from a different region of cloth (3 total), were taken from shirts A, B, and C, and analyzed for isotopic signatures.

#### 2.5.4 T-Shirt Homogeneity

Three samples were taken from four different regions of shirt (front and back panels, left and right sleeves) for a total of 12 samples per shirt. This process was repeated for shirts A, B, and C to test for homogeneity of different regions of the shirts.

Each of the procedures outlined above for the four experiments (2.5.1–2.5.4) were repeated twice; once for ^13^C isotopic analysis and a second time for ^2^H and ^18^O stable isotope analysis.

### 2.6 Data Compilation and Analysis

Data was collected across multiple days to ensure reproducibility of results. Raw data was obtained from the proprietary software (Isodat 3.0) and exported to Microsoft Excel. These values were then normalized using the standards for each stable isotope, and averaged to obtain a representative δ-value. After determination of averages and standard deviations, ANOVA tests (at the 95% confidence level) were performed to determine p-values demonstrating the differentiability of each sample. Data was compiled into a three-dimensional trivariate plot in Wolfram Mathematica for visualization. Averages were treated as points with the standard deviations for each of the three isotopes extended in the *x*, *y*, and *z* directions and covered in a surface, creating an ellipsoid around the central average.

## 3 Results and Discussion

### 3.1 Eight Cotton Types

δ^13^C values range from −27.2 to −25.0, while δ^2^H and δ^18^O values varied more, ranging from −64.7 to −12.0 and 21.9 to 33.8, respectively (Table 1). *P*-values show that all eight types of cotton are statistically different in at least one of their δ^13^C, δ^2^H, or δ^18^O values (Table 2). In many cases, δ^2^H values seem to be the least useful due to their large standard deviation, but are still suitable for differentiation in the majority of cases.

**Table 1:** Average ± Standard Deviations of δ^13^C, δ^18^O, and δ^2^H Values (‰) for Eight Cotton Samples (*n* = 12)

sample	δ^13^C	δ^18^O	δ^2^H
shirt A	–26.2 ± 0.2	30.3 ± 0.7	–37 ± 7
shirt B	–25.0 ± 0.1	31.2 ± 0.6	–31 ± 5
shirt C	–24.1 ± 0.2	24.7 ± 0.2	–41 ± 2
towel A	–27.2 ± 0.2	26.1 ± 0.9	–54 ± 3
towel B	–25.1 ± 0.1	28.1 ± 0.7	–37 ± 3
cloth	–25.6 ± 0.2	33.8 ± 0.7	–12 ± 4
gauze A	–26.3 ± 0.2	26 ± 1	–41 ± 3
gauze B	–26.8 ± 0.1	22 ± 1	–65 ± 3

**Table 2:**
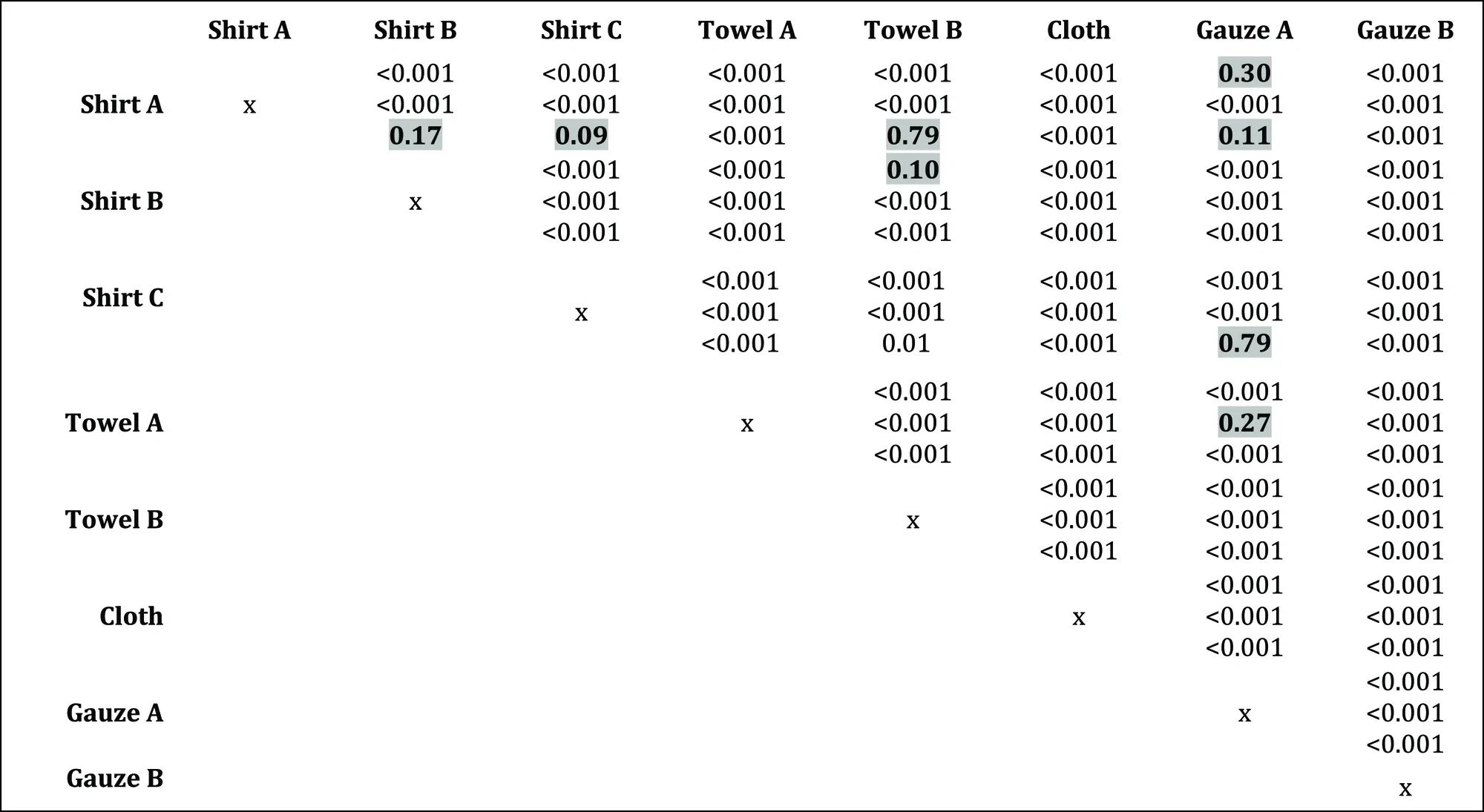
*P*-Values from ANOVA Tests Showing Differentiability of Eight Cotton Samples^a^

a The values at the top, middle, and bottom of each cell are for δ^13^C, δ^18^O, and δ^2^H values, respectively. Bolded and highlighted values are higher than a *p*-value of 0.05 and are non-differentiable at the 95% confidence level.

A three-dimensional scatter plot shows the clear differentiability of the data (Figure 1). The space filled by each of the eight different types of cotton is isolated from all sides, demonstrating the unique isotopic signature of each sample. Furthermore, rotation of the plot in three dimensions reveals the isotopic differences between each type of cotton even more clearly. Such clear differentiability between each of the samples tested is of significant forensic importance, as a fiber with a given isotopic signature may be correlated to a source with a significant level of certainty in each of our samples. Further analysis of a larger cohort of sources would demonstrate if this finding is generally applicable to all cotton shirts and other products.

**Figure 1:**
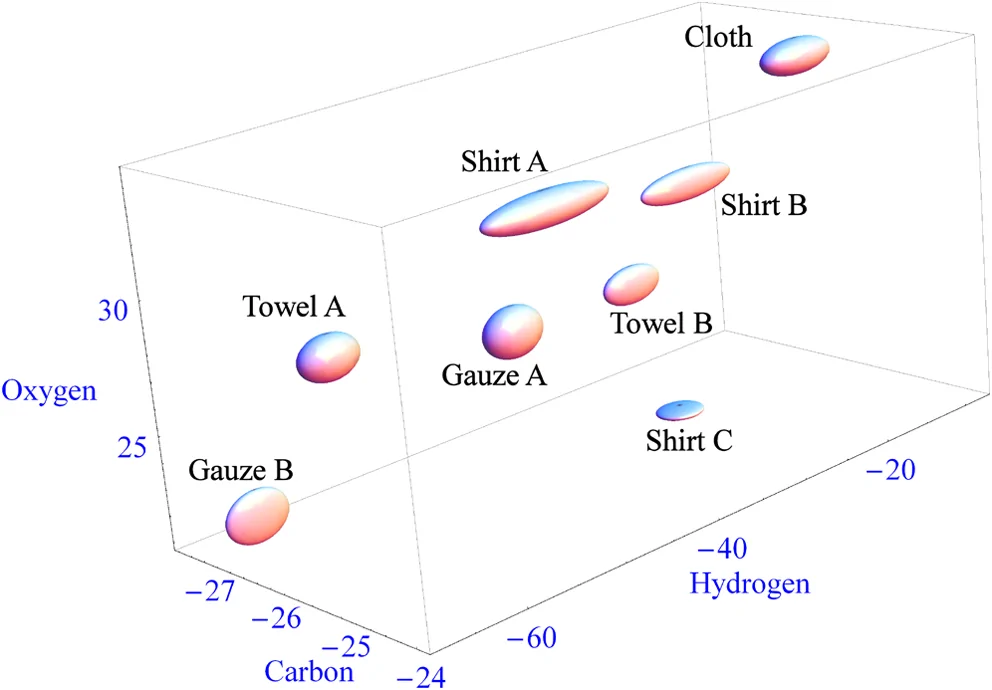
Three-dimensional scatter plot showing differentiability of eight cotton types.

### 3.2 T-Shirts of Identical Origins

When three t-shirts from the same package were analyzed, the δ^13^C, δ^2^H, and δ^18^O values for all three shirts were statistically indistinguishable (Table 3). Standard deviations were also extremely low, indicating the homogeneity within shirts of identical origins. Presumably, shirts that are packaged together were manufactured by the same company in the same location at the same time, with cotton from the same source, leading to the observed similarities. From a forensic standpoint, these results could allow for the testing of any shirt that comes from the same suspected batch as the crime scene sample. This ability could prove to be a useful tool in the event a suspect has disposed of the original shirt in an effort to conceal evidence, since other shirts from the same packaging could equally demonstrate a potential link between suspect and crime.

**Table 3:** Average δ-Values of Three Undershirts from the Same Package Showing Homogeneity (*n* = 6)

		average ± St. Dev.
shirt 1	δ^13^C	–24.2 ± 0.2
	δ^18^O	24.6 ± 0.2
	δ^2^H	–40.3 ± 1.8
shirt 2	δ^13^C	–24.1 ± 0.2
	δ^18^O	24.7 ± 0.1
	δ^2^H	–40.4 ± 2.3
shirt 3	δ^13^C	–24.1 ± 0.3
	δ^18^O	24.7 ± 0.2
	δ^2^H	–41.5 ± 1.2

### 3.3 Sewing Thread Versus Cloth Fiber

Sewing thread and cloth fibers are differentiable for all three shirts tested (Table 4). This presumably arises from the use of different cotton sources for cotton thread and cotton cloth. Such distinct variation between the two parts of the shirt illustrates the importance of testing both sewing thread and fiber when carrying out forensic analysis. Careful analysis of both types of fiber would help to avoid a false-negative correlation originating from a δ-value from the wrong component of the shirt.

**Table 4:** Delta Values of Sewing Thread and Cloth Fibers from Three T-Shirts^a^

	shirt A			shirt B			shirt C		
	δ^13^C	δ^18^O	δ^2^H	δ^13^C	δ^18^O	δ^2^H	δ^13^C	δ^18^O	δ^2^H
sewing thread	–25.3 ± 0.1	11.3 ± 0.7	–71 ± 2	–24.7 ± 0.1	11.0 ± 0.4	–68 ± 4	–24.9 ± 0.1	21.9 ± 0.2	–76 ± 2
cloth fiber	–26.2 ± 0.2	30.3 ± 0.7	–37 ± 7	–25.0 ± 0.1	31.2 ± 0.6	–31 ± 5	–24.1 ± 0.2	24.7 ± 0.2	–41 ± 2
*P*-value	<0.001	<0.001	<0.001	<0.001	<0.001	<0.001	<0.001	<0.001	<0.001

a *P*-values show the differentiability between the two types of fiber (*n* = 9).

A three-dimensional scatter plot illustrates the differentiability of sewing thread and the cloth fibers from each shirt (Figure 2). It also reveals that the fibers are grouped tightly in very different regions of the plot, with the sewing thread in the bottom left at low δ^2^H and δ^18^O values, and the cloth fibers grouped in the upper right at higher δ^2^H and δ^18^O values. This is not surprising as sewing thread is often made from synthetic material or a cotton-synthetic blend due to increased strength of synthetic materials.

**Figure 2:**
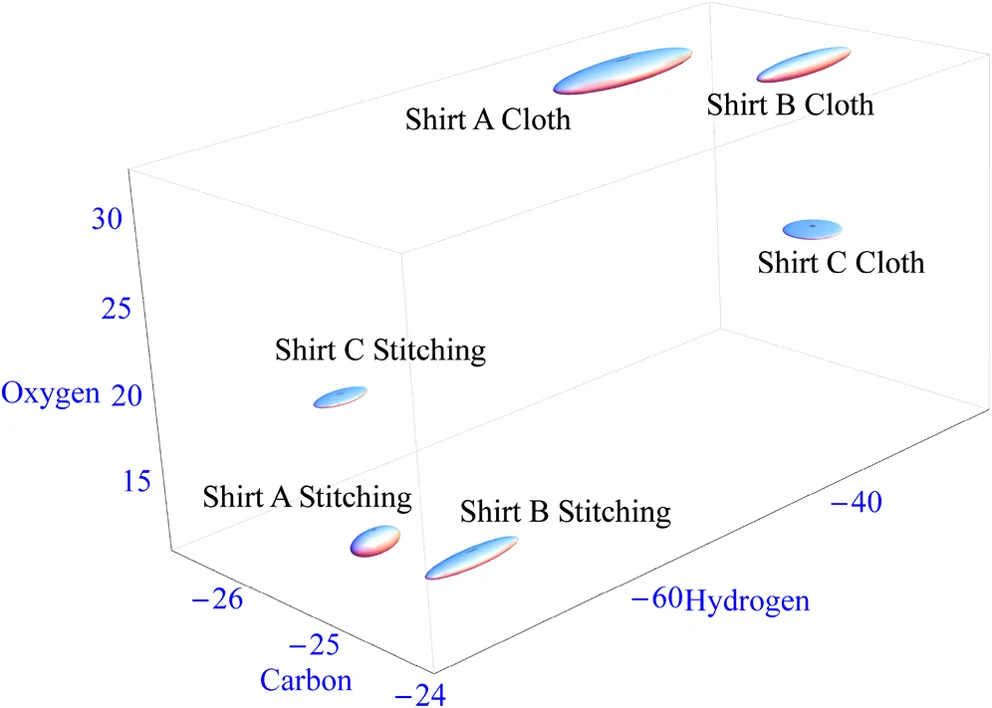
Three-dimensional scatter plot showing differentiability and grouping of sewing thread/stitching and cloth fibers.

### 3.4 T-Shirt Homogeneity

All three shirts show almost complete homogeneity in all three stable isotopes. This demonstrates the homogeneity of the cotton material used in the shirt manufacturing process, which is important for the matching of a fiber found at a crime scene to a shirt in the possession of a suspect. However, one shirt exhibited isotopic differences between the back panel and the two sleeves (Table 5). This discrepancy between different parts of the shirt could arise from different bolts of cloth being used for each part of the shirt, with the various parts later stitched together. This implies that in an IRMS forensic investigation, fiber samples from each separate part of the shirt (front panel, back panel, sleeves, pocket, etc.) should be analyzed and the stable isotope ratios of each part compared to the evidence in question.

**Table 5:** *P*-Values for δ^13^C from Shirt B Showing Statistical Differences between the Back Panel and other Portions of the Shirt^a^

shirt B	left sleeve	right sleeve	front	back
left sleeve	x	**0**.**13**	**0**.**43**	<0.001
right sleeve		x	**0**.**49**	<0.001
front			x	<0.001
back				x

a Bolded and highlighted values are higher than a *p*-value of 0.05, and are therefore statistically indistinguishable at the 95% confidence level.

## 4 Conclusions

In this initial study, we investigated the potential for using stable isotope ratio mass spectrometry for forensic analysis of cotton fibers originating from t-shirts and other common cotton products. We found δ^13^C, δ^2^H, and δ^18^O values to all be useful in this analysis. We demonstrated the differentiability of eight cotton samples based solely on isotopic signatures. We also showed the homogeneity of sources from identical origins, and determined that sewing thread and cloth from the same shirt have distinct isotopic signatures as might different cloth sections of the shirt. These last findings demand caution for forensic investigations, as different fibers from a shirt could lead to an erroneous negative correlation between suspect and crime. With the relatively small number of samples included in this preliminary study, care must be taken to with respect to the ability to extrapolate results to all fabric samples. Future studies should incorporate larger numbers of fabrics in order to increase the statistical power of the presented method. Dyes and other cloth treatments should also be investigated to determine their effects on stable isotope ratios. Finally, cotton-polyester and other cotton-cloth blends should be studied to establish the effects of other fibers on the stable isotope ratios and differentiability of samples.
